# SOCIOECONOMIC AND NUTRITIONAL CHARACTERISTICS OF CHILDREN AND ADOLESCENTS WITH SICKLE CELL ANEMIA: A SYSTEMATIC REVIEW

**DOI:** 10.1590/1984-0462/;2018;36;4;00010

**Published:** 2018

**Authors:** Amanda Cristina da Silva de Jesus, Tulio Konstantyner, Ianna Karolina Véras Lôbo, Josefina Aparecida Pellegrini Braga

**Affiliations:** a Department of Pediatrics, Universidade Federal de São Paulo, São Paulo, SP, Brazil.

**Keywords:** Sickle cell anemia, Child, Adolescent, Social class, Anthropometry, Anemia falciforme, Criança, Adolescente, Classe social, Antropometria

## Abstract

**Objective:**

To describe the socioeconomic and nutritional characteristics of children and adolescents with sickle cell anemia.

**Data sources:**

The present study is a systematic literature review based on published scientific articles. The searches were carried out using the electronic database of the National Library of Medicine, National Institutes of Health- PubMed. Two searches of articles published in the last 20years and without limitation of language were carried out. Thefirst one started from the Medical Subject Headings term “Anemia, Sickle Cell” associated with “Socioeconomic Factors”; and the second started from the term “Anemia, Sickle Cell” associated with “Anthropometry”. Thesearches were directed to research conducted on humans in the age group from 0 to 18years.

**Data synthesis:**

The final selection was composed by 11 articles on socioeconomic characteristics and 21articles on nutritional characteristics. Allstudies included children and adolescents with sickle cells disease (age range 0-18years), both genders, and most of them of black ethnicity. Families of children and adolescents with sickle cell anemia were of predominantly low socioeconomic status. Parents had lower educational levels when compared to parents of healthy children and adolescents. Body measurements (weight and height) and anthropometric indicators of children with sickle cell anemia were often lower when compared to healthy groups or reference populations.

**Conclusions:**

Children and adolescents with sickle cell anemia have socioeconomic limitations and worse nutritional conditions, when compared to reference populations. These limitations may lead to worse growth and greater occurrence of possible complications that can impair their quality of life.

## RESUMO

**Objetivo:**

Descrever as características socioeconômicas e nutricionais de crianças e adolescentes com anemia falciforme.

**Fonte de dados:**

Estudo de revisão sistemática da literatura baseado em artigos publicados em revistas científicas. As buscas foram realizadas na base de dados eletrônica da *National Library of Medicine, National Institutes of Health- PubMed.*Foram realizadas duas buscas de artigos publicados nos últimos 20anos e sem limitação de idioma. Aprimeira partiu do Descritor em Ciências da Saúde “Anemia Falciforme” associado com “Fatores Socioeconômicos”; e a segunda partiu do descritor “Anemia Falciforme” associado com “Antropometria”. Asbuscas foram direcionadas para pesquisas realizadas em seres humanos na faixa etária de 0 a 18anos.

**Síntese dos dados:**

A seleção final foi composta por 11artigos referentes às características socioeconômicas e 21 referentes às características nutricionais. Asamostras estudadas foram de crianças e adolescentes de ambos os sexos, com idade de 0 a 18anos e com predomínio de populações negras. Famílias de crianças e adolescentes com anemia falciforme eram predominantemente de baixo nível socioeconômico. Ospais apresentaram níveis educacionais inferiores, quando comparados a pais de crianças e adolescentes saudáveis. Asmedidas corporais (peso e estatura) e os indicadores antropométricos de crianças com anemia falciforme foram frequentemente menores, quando comparados com os dos grupos saudáveis ou das populações de referência.

**Conclusão:**

Crianças e adolescentes com anemia falciforme apresentam limitações socioeconômicas e piores condições nutricionais, quando comparados às populações de referência. Essas limitações podem implicar pior crescimento e maior ocorrência de possíveis complicações, que podem prejudicar sua qualidade de vida.

## INTRODUCTION

Sickle cell disease (SCD) occurs due to a genetic mutation- replacement of the nitrogen base thymine with adenine, which results in the substitution of glutamic acid with valine at the sixth position of the β chain on the short arm of chromosome 11. This change leads to formation of structurally abnormal hemoglobin, called hemoglobin S (HbS), and, consequently, deformation and stiffening of the red cell membrane.

In Brazil, an estimated 3,000 children are born with SCD annually. The term “sickle cell anemia” (SCA) is used for its form of homozygotes SS, being characterized as one of the most common hereditary diseases in different populations.^1^ ^,^ ^2^ ^,^ ^3^ SCD is considered a public health problem with a high prevalence among people of black ethnicity, who in many cases make up the poorest groups in society, live in peripheral regions of large urban centers and have less access to health and education.^4^ ^,^ ^5^

In most cases, clinical manifestations of SCA begin in early childhood with significant nutritional and psychosocial impact.^2^ The growth and development patterns present differences in children and adolescents with and without SCA in all age groups. Often, the weight and height of children and adolescents with SCA are lower when compared to children and adolescents without the disease. These differences have been associated with higher total energy expenditure, lower circulating hemoglobin levels, and higher frequency of hospitalizations of patients with SCA.^2^ ^,^ ^3^ ^,^ ^6^

As in other chronic diseases, low socioeconomic and educational levels directly affect the quality of life of these children and adolescents with SCA. These characteristics are associated with a worse prognosis, since its impact is multifactorial and directly interferes with nutrition and health care.^3^ ^,^ ^7^ ^,^ ^8^

The difficulty of parents and children with SCA in dealing with the need imposed by the consequences of the disease, such as repetitive infections, pain, blood transfusions and frequent hospitalizations, which result in absenteeism and lower school performance, highlights the importance of ensuring adequate nutritional support and the existence of minimum socioeconomic conditions for clinical and family management. However, there is still a lack of studies that broadly describe the socioeconomic (SC) and nutritional (NC) characteristics of children and adolescents with SCA.^4^ ^,^ ^9^

In this context, this study aimed to describe the SC and NC of children and adolescents with SCA in different populations, offering a broad epidemiological scenario to contribute to the elaboration and execution of management and control strategies for the clinical consequences of the disease.

## METHOD

The present study is a systematic literature review based on articles published in scientific journals. The searches were conducted in the electronic database of the National Center for Biotechnology Information Advances Science and Health- National Library of Medicine- National Institutes of Health-PubMed,^10^ in March 24^th^ 2017.

Two searches were carried out to meet the proposed objectives without limitation of language. The health descriptors were chosen by consulting Medical Subject Headings (MesH).Thesearch for SC came from the descriptor “*anemia, sickle cell*” associated (*and*) with “*socioeconomic factors*”, which resulted in 296 articles. The search for NC was based on the descriptor “*anemia, sickle cell*” associated (*and*) with “*anthropometry*”, which resulted in 288 articles. In both searches, filters were added for research done on humans (*humans*) and on children and adolescents (*child: birth 18 years*).

After applying the filters, the search for SC resulted in 194 articles, and that for NC resulted in 221 articles. These articles were evaluated for their methodological characteristics, excluding those which studied subjects without SCA, studies conducted predominantly with adults (sample of individuals aged over 18 years greater than 80%), studies with samples composed of pregnant women or patients with SCA associated with other hemoglobinopathies or other diseases, articles published over than 20 years ago, review articles, duplicate articles, letters to the editor, and case reports. Thus, 72 articles on SC and 94 on NC remained. Given the changes in disease control related to better resources for early diagnosis and targeted care, articles published over than 20 years ago were excluded, as it was considered that the results possibly found would not reflect the nutritional and social reality of children and adolescents with SCA receiving care in today’s world.

The abstracts of these articles were read independently by two researchers, who excluded those who did not present information to answer the research questions (socioeconomic factors and anthropometric data with a sample of children and adolescents with SCA).

After this stage, the researchers gathered in a panel for discussing the eligibility criteria, observing agreements and disagreements in the choices. Thus, agreeing options on inclusion or exclusion were maintained, and disagreements were re-evaluated together in the presence of a third investigator, resulting in 11 articles from the search for SC and 23 for NC.

The SC found in the selected articles were socioeconomic classifications (estimated by several criteria at different class levels) and specific indicators (parents’ occupation and schooling, insurance/health insurance and family income).

At the same time, the NCs found were weight (W), height (H), absolute body mass index (BMI) and anthropometric indicators of weight/age (W/A), weight/height (W/H), height/age (H/A) and body mass index/age (BMI/A). The population reference standards considered were: World Health Organization (WHO),^11^ Centers for Disease Control and Prevention(CDC)^12^ and National Center for Health Statistics(NCHS).^13^ In addition, healthy control group studies were also included.

Subsequently, 3 of the 23 articles selected in the NC group were excluded because they did not present consistent information on the nutritional status of the children and adolescents studied. In addition, an article from the SC search that went through this stage also contained nutritional information, and was therefore included in the NC search.

**Figure 1:**
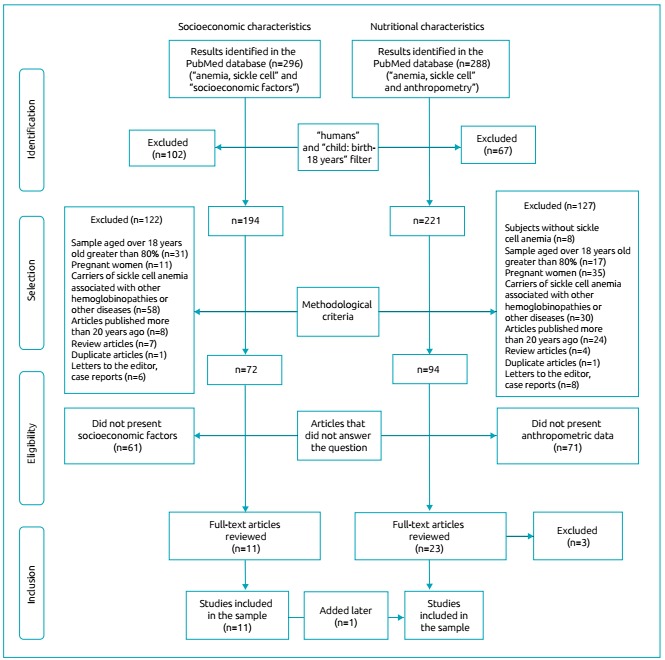
Selection process of studies on socioeconomic and nutritional characteristics of children and adolescents with sickle cell anemia (aged 0 to 18 years), 1996 to 2017.

Finally, 11 articles on SC and 21 on NC were selected and analyzed in the present study. The selection process of the articles is presented in Figure 1.

## RESULTS

The methodology used to search for information resulted in the selection of 11 articles referring to SC and 21 referring to NC, most of which were carried out in clinics and hospitals from reference centers and in universities in cities of the United States and Nigeria. The samples studied were children and adolescents with SCA of both sexes, aged between 0 and 18 years and with predominance of black populations.

In all studies, interviews were conducted with parents or guardians. The SCs were evaluated by the parents’ schooling and their occupations, the family income and the application of specific indexes and classifications. On the other hand, NCs were evaluated by W, H, and anthropometric indices, compared with the study’s own control groups or with population reference standards (Tables1 and 2).

**Table 1:** Socioeconomic characteristics of children and adolescents (aged 0 to 18 years) with sickle cell anemia, based on studies published between 1996 and 2017.

Author (year)	Methodology				Results
	Design	Sample	Place (city/state/county)	Evaluation criteria*	
Fernandes etal*.* (2015)^14^	T	n=106 (45 ♀/61 ♂) 8-14 y	Minas Gerais, Brazil	Parental schooling (≤8 years of schooling) x̄ annual FI (in thousands of USD) by age group and home ownership	Maternal schooling (74.2%); paternal schooling (75.4%) Annual FI:8-10 (7.0); 11-14 (17.5%) Home ownership:No (68.5%)
Kingetal*.* (2014)^15^	T	n=107 SS/43 AA 5-15 y	USA	Schooling of the head of the family and annual FI (in thousands of USD)	Some schooling or more: SS (57.0%); AA (60.5%) Annual FI:SS (8.4); AA (11.0)
Ezenwosu etal*.* (2013)^16^	CC	n=90 SS/90 AA (35 ♀/55 ♂) 5-11 y	Enugu, Nigeria	SC (parental/guardians’ occupation and schooling)	SS SC: high (30%); average (23.3%); low (46.7%) AA SC: high (23.3%); average (27.8%); low (48.6%)
Akoduetal*.* (2012)^17^	T	n=80 SS/80 AA 2-15 y	Lagos, Nigeria	SL and socioeconomic stratum	SS SL: I-II (36.3%); III-IV (62.5%); V (1.2%) AA SL: I-II (51.3%); III-IV (48.7%); V (0%) SS stratum: high (36.2%); average (45%); low (18.8%) AA stratum: high (51.2%); average (38.8%); low (10%)
Lunaetal*.* (2012)^18^	T	n=160; 3-12 y	Pernambuco, Brazil	Parents’ combined educational level and FI (BMW)	Schooling: I-L (5%); IPE (56.3%); CPE (18.1%); CSE (18.1%); HE (2.5%) FI: <1 BMW (15%); 1-2 BMW (76.9%); >2 BMW (7.5%); ND (0.6%)
Boulet etal*.* (2010)^19^	T	n=192 SS/19,335 AA 0-17 y	NHIS - USA	Maternal schooling (ISE, CSE and HE), FI (below the FPI) and insurance (health insurance - Medicait/SCHIP)	SS schooling: ISE (28%); CSE (32.4%); HE (39.6%) AA schooling: ISE (19%); CSE (33%); HE (48%)FI<FPI: SS (47.8%); AA (34.7%); p*<0.05* Insurance: SS (56.2%); AA (39.7%); p*<0.05*
Brown etal*.* (2010)^20^	T	n=67 (24 ♀/43 ♂) 0-18 y	Ibadan, Nigeria	Maternal schooling	Maternal schooling: ≥SE (50.7%); <SE (49.3%)
Uchendu etal*.* (2010)^21^	CC	n=100 (♂); 6-18 y	Enugu, Nigeria	SC (parental/guardians’ occupation and schooling)	SC: high (19%); average (30%); low (51%)
Panepinto etal*.* (2009)^22^	T	n=104; 2-18 y	Wisconsin, USA	FI (in thousands of USD)	FI: >40 (21.2%); >20 to ≤40 (25%); ≤20 (34.6%); ND (19.2%)
Telfairetal*.* (2003)^23^	T	n=662	Alabama, USA	x̄ CSI	CSI: urban (11.97); rural (13.9)
Singhal etal*.* (1996)^24^	Co	n=219 (106 ♀/113 ♂) 0-9 y	Kingston, Jamaica	x̄ SC score ^†^	SC: ♀ (11); ♂ (12)

T: transversal; CC: case-control; Co: cohort; y: years; SS: children with sickle cell anemia; AA: healthy children; SC: socioeconomic condition; SL: socioeconomic level; BMW: Brazilian minimum wage; x̄: average; FI: Family income; NHIS: National Health Interview Survey ; I: illiterate; L: literate (out of school); IPE: incomplete primary education; CPE: complete primary education; ISE: incomplete secondary education; CSE: complete secondary education; HE: higher education; ND: not declared; FPI: federal poverty index; Medicait/SCHIP: health care program for families with lower income in the USA; CSI: Community Stress Index (poverty, schooling, ≥ 16 years of age who cannot work, ≥ 16 years of age who are unemployed and income - scoring ranges from 1 to 18 points; stress levels are high ≥ 14, average from 9 to 13 and low from 0 to 8); ^†^measured by the number and types of household appliances, water source and type of sanitation (scale from 1 to 18); *SC information was collected through an interview with parents or guardians.

**Table 2:** Nutritional characteristics of children and adolescents (aged 0 to 18 years) with sickle cell anemia, based on studies published between 1996 and 2017.

Author (year)	Methodology				Results
	Design	Sample	Place	Evaluation criteria	
Orimadegun & Onazi (2015)^25^	T	n=208 (76 ♀/132 ♂); 9 m-15 y	Zamfara, Nigeria	W (kg), BMI (kg/m²) and W/H (Z<-2) (WHO)	W=9.0±7.2; BMI=14.3±2.2; W/H=24.5%
Akoduetal*.* (2014)^26^	CC	n=50 SS/50 AA (50 ♀/50 ♂); 9 m-15 y	Lagos, Nigeria	x̄ H (cm)	H: SS 113.3/AA 115.4*
Salles et al.*.* (2014)^27^	Co	n=76 (non-apneic); x̄=9±3 y	Salvador, Brazil	x̄ H/A and BMI/A (Z/CDC)	H/A=-0.7 (-1.4/-0.1); BMI/A=-1.0 (-2.2/-0.2)
Akodu etal*.* (2012)^17^	CC	n=40 SS/40 AA; 2-15 y	Lagos, Nigeria	x̄ BMI/A (Z-WHO)	♀ SS -1.72/AA 0.03; p<0.001; ♂ SS -1.1/AA -1.01*
Animasahun etal*.* (2011)^28^	CC	n=100 SS/100 AA; 1-10 y	Lagos, Nigeria	H (m), W (kg) and BMI (kg/m²)	H: SS 1.13/AA 1.25*;W: SS 18.8/AA 21.3; p<0.001 BMI: SS 14.6/AA 15.1*
Wali & Moheeb (2011)^29^	CC	n=41 SS/50 AA (♂); 10-14 y	Muscat, Oman	x̄ W (kg) and H (cm)	W: SS 31.7/AA 33.6*; H: SS 142.2/AA 144.6*
Al-Saqladietal*.* (2010)^30^	T	n=102	Aden, Yemen	x̄ H/A, W/A, W/H and BMI/A (Z-WHO)	≤5y: H/A=-2.16; W/A=-2.17; BMI/A=-1.21 >5y: H/A=-2.24; W/A=-2.68; BMI/A=-1.84
Sadarangani etal*.* (2009)^31^	Co	n=124 (56 ♀/68 ♂); 0-18 y	Kilifi, Kenya	M_e_ H/A, W/A and W/H (Z-NCHS)	H/A=-1.90; W/A=-2.00; W/H=-1.50
Fung etal*.* (2008)^32^	Co	n=80 (40 ♀/40 ♂); 4-19 y	Philadelphia, USA	H/A, W/A and BMI/A (Z-WHO)	♀ H/A=-0.1; W/A=-0.5; BMI/A=-0.6 ♂ H/A=-0.9; W/A=-1.2; BMI/A=-0.9
Koumbourlis & Lee (2007)^33^	Co	n=45 (25 ♀/20 ♂); 10.6±3.5	Colombia & USA	H/A, W/A and BMI/A (percentile-NCHS)	H/A=35; W/A=40; BMI/A=45
Kwachaketal*.* (2007)^34^	Co	n=97 (53 ♀/44 ♂); 1-18 y	Philadelphia, USA	x̄ H/A, W/A and BMI/A (Z-CDC)	H/A=-0.5; W/A=-0.8; BMI/A=-0.7
Zemel etal*.* (2007)^35^	Co	n=578 (278 ♀/300 ♂); x̄=9.1±4.7y	Philadelphia, USA	x̄ W/A, BMI/A (Z-CDC)	W/A=-0.7*; BMI/A=-0.7*
Buison etal*.* (2005)^36^	T	n=90 SS/198 AA; 4-19 y	Philadelphia, USA	x̄ H/A, W/A and BMI/A (Z-CDC)	H/A: SS -0.46/AA 0.27;p<0.001 W/A: SS 0.80/AA 0.39;p<0.001 BMI/A: SS -0.77/AA 0.29;p<0.001
Barden etal*.* (2002)^37^	CC	n=36 SS/30 AA; 5-18 y	Philadelphia, USA	x̄ H/A, W/A (Z-NCHS)	H/A: SS -0.4/AA 0.5;p<0.01; W/A: SS -0.8/AA 0.3; p<0.01
Buchowski etal*.* (2002)^38^	CC	n=37 SS/23 AA; 14-18 y	Tennessee, USA	x̄ H (cm) and H (kg)	♀ H: SS 154.4/AA 163.8*; W: SS 50.8/AA 59.2* ♂ H: SS 167.0/AA 163.8*; W: SS 55.8/AA 53.1*
Oredugba etal*.* (2002)^39^	CC	n=117 SS/122 AA; 10±4.7 y	Lagos, Nigeria	H (m) and W (kg)	<6 y H: SS 0.94/AA 0.98*; W: SS 13.8/AA 15.6* 6-12 y H: SS 1.46/AA 1.50*; W: SS 31.0/AA 35.3* >12 y H: SS 1.64/AA 1.62*; W: SS 47.2/AA 55.1;p<0.01
Singhal etal*.* (2002)^40^	CC	n=41 SS/31 AA; 3-6 y	Kingston, Jamaica	x̄ H (cm), W (kg), BMI (kg/m²)	♀ H: SS 107.9/AA 109.8*; W: SS 16.6/AA 17.6* BMI: SS 14.2/AA 14.5* ♂ H: SS 105.8/AA 107.9*; W: SS 16.3/AA 17.2* BMI: SS 14.5/AA 14.7*
Cipolotti etal*.* (2000)^41^	T	n=76 (42 ♀/34 ♂)	Sergipe, Brazil	x̄ H/A and W/A (Z-NCHS)	<15 y H/A-W/A=NCHS*; ≥15 y H/A-W/A <NCHS (95%CI)
Thomasetal*.* (2000)^42^	Co	n=315; 0-18 y	Kingston, Jamaica	x̄ H/A and W/A (Z-NCHS)	♀ H/A=-1.4; W/A=-1.7 ♂ H/A=-1.7; W/A=-1.7
Kopp-Hoolihan etal*.* (1999)^43^	T	n=8 (3 ♀/5 ♂); 11-18 y	California, USA	x̄ H/A, W/A (Z-NCHS) and BMI	E/I=-1.5; W/A=1.3; BMI/A=17.9
Soliman etal*.* (1998)^44^	CC	n=162	Muscat, Oman	H/A=-1.52; BMI=14.6	H/A=-1.52; BMI=14.6

T: transversal; CC: case-control; Co: cohort; m: XXXXX; y: years; x̄: mean; M_e_: median; W: weight; H: height; A: age (months); BMI: body mass index; Z: z score; SS: children with sickle cell anemia; AA: healthy children; WHO: World Health Organization; CDC: Centers for Disease Control and Prevention; NCHS: National Center for Health Statistics; *p>0.05; 95%CI: 95% confidence interval.

In the studies that presented the SCs, the children and adolescents with SCA belonged to families with socioeconomic conditions between middle and low level, and most of the parents had some type of schooling, predominating between primary and secondary education. Although the SCs of the group of children and adolescents with SCA presented lower levels, only one study compared them with the control group, showing, in a significant way, that children with SCA belonged to families of lower income and with greater need of use of the public health care network (Table1).

In relation to NCs, the measurements and the anthropometric indexes of children and adolescents with SCA showed statistically significant lower values for the control and/or reference populations in 5 of the 21 selected studies (Table2).

## DISCUSSION

The results show that children and adolescents with SCA predominantly belong to families from the less favored socioeconomic classes, whose parents have greater difficulty to achieve higher educational levels when compared to parents of healthy children and adolescents.

Socioeconomic status is a factor that directly affects treatment and access to health care. Adherence and attendance to follow-up visits are often affected by the low family income, especially by the distance between the home and the specialized services facilities. This phenomenon is aggravated when it comes to patients with chronic disease in a situation of dependence, as is the case of children with SCA.^19^

Because it is a hereditary disease, descended from sub-Saharan African populations,^5^ SCA is embedded in a context of social inequality in the world. The selected studies, for the most part, were performed in samples exclusively composed or with a large percentage of black population.

According to the Brazilian Institute of Geography and Statistics (IBGE),^45^ the majority of the Brazilian population (53.9%) declared to be of black or brown color/race, and this characteristic is higher in the age group from 10 to 19 years (values greater than 58.0% in both sexes). In addition, data show that, in relation to food unsafety (mild, moderate and severe), black children are the most exposed to this nutritional risk (43.1%). Access to basic sanitation services (water supply by general network, sanitary sewage by collecting or rainwater network, and direct or indirect garbage collection) is also lower in the black population, with only 55.3% of households in this group presenting this characteristic, different from the white population (71.9%).

Historically, the black population became enslaved in Brazil and, even after the abolition of slavery, their descendants had few opportunities for social and economic entry into the country. An improvement can be observed in the living conditions of black populations in Brazil, but this socioeconomic structural problem remains today.^46^

The sum of these problems implies worse socioeconomic situations. An example of this is the low level of education of children’s guardians, which makes it difficult to get a formal job. Thus, families are more inclined to work with informal and uncertain services, without the security of a monthly income.^47^ This aspect is also reflected in the schooling of children with SCA from families with low socioeconomic status, who have lower school performance, with little possibility of access to reinforcement activities or other type of support that contributes to performance improvement.^16^

Thus, children with SCA, which predominantly comprise the black population by the disease’s inheritance characteristic, are more likely to live in worse socioeconomic conditions. In this sense, it seems plausible that the Brazilian data can be extrapolated to countries with similar ethnic characteristics, which present different degrees of miscegenation, but which predominantly have the worse living conditions among the black population, when compared to the white population.

At the same time, it was possible to identify in the articles selected in the present review that the body measurements (Wand H) and the anthropometric indicators of children with SCA were often lower when compared with reference populations (WHO, CDC and NCHS) and with control groups consisting of children and adolescents.

Thus, SCA represents a greater risk of interrupting W and H gain and, consequently, leads to chronic protein and energy malnutrition. When compared to the healthy population, the basal metabolic rate of children with SCA is 16 to 20% higher. Impaired nutritional status can bring even more important clinical complications to children with this disease, negatively affecting the individual’s health and nutrition, resulting in an unwanted cycle of health problems.^48^

In fact, there were statistically significant differences inW when children with SCA were compared to childrenin the control groups. Such difference was not evidenced inthe comparison of H. However, the mean H of these children was lower when compared to the population reference curves. This can be explained by the fact that the indicators adopted were developed based on different populations, including several races, social classes and living conditions, which present higher means when compared to other less favored populations such as children with SCA in Nigeria and Jamaica.^28^

Low H and the slower growth rate of children with SCA have been associated with delayed epiphyseal fusion of long bones, due to late puberty, and the lower concentration of circulating hemoglobin. Although the end of the growth period of these children occurs somewhat later than with healthy children, the final H cannot compensate for the previous delay and therefore is compromised, not reaching the expected height for a healthy individual.^41^ ^,^ ^49^ Another review of the growth and nutritional status of children and adolescents with SCA, published in 2008, selected 46 studies, but with large variations in sample size and reference standards, which reduced the ability to compare them. However, as demonstrated in the present study, there was a consistent pattern of growth failure among affected children in all geographic areas, with good evidence linking growth failure to endocrine dysfunction, metabolic disorders, and specific nutrient deficiencies.^50^

On the other hand, recent studies have identified an increase in the prevalence of excess W in children with SCA. Such evidence may be associated with better control of the disease and the period of nutritional transition observed worldwide in the last years with the exposure of children to a large obesogenic environment. Akodu etal*.*,^17^ in a study with 80 children with SCA, showed that 2.5% were obese, an estimate that had not been identified until that date in the global society. This finding raises the possibility of the beginning of a period of changes in the anthropometric pattern of children and adolescents with SCA in the world, who would be closely related to the populations’ socioeconomic and cultural characteristics, which, in turn, correlate directly with nutritional status, since they are determinants of adequate physical growth, especially for children and adolescents with this chronic disease.^28^

It should be noted that the systematic review carried out in this study was based on a rigorous research methodology in the scientific literature, which minimizes the loss of information on the two topics studied. However, it was decided to use articles only from the last 20 years, considering that older articles would not bring real data on the current situation of the SC and NC of children with SCA.

In the context of SCs and NCs, children and adolescents with SCA and their families seem to depend on improved access conditions to specialized health centers, especially those residing in remote areas, to receive adequate care and treatment. Access to health guidance and resources for nutritional support and disease management is paramount for proper physical development and maintenance of health status.

However, hereditary hemoglobin disorders, such as SCA, have become an increasingly neglected global health burden and a challenge for health managers and professionals. Babies who had previously died of this disease, before being recognized, began to live to diagnosis and treatment as developing countries undergo the epidemiological transition and improve the living conditions of their populations.^51^ ^,^ ^52^ Especially in developing countries, one of the major challenges is the scarcity of laboratory infrastructure to test SCA. For the adequate control of the socioeconomic impact and the clinical and nutritional consequences, a cheap and rapid diagnostic test that can identify the disease early in environments with few resources is necessary.^53^ ^,^ ^54^ In this context, the results of this study suggest that children and adolescents with SCA present socioeconomic limitations and worse anthropometric and nutritional conditions when compared to reference populations. Such evidence is aggravated by the direct association between socioeconomic and nutritional conditions, which implies a worse growth of these children and a higher occurrence of possible complications that may impair their quality of life.

Thus, health strategies directed at children and adolescents with SCA should include monitoring of growth and nutritional status as an essential requirement for comprehensive care, facilitating the diagnosis of growth failure and early nutritional intervention. Such strategies should consider the socioeconomic status of their families as an alternative to control and prevent unwanted clinical consequences.

Finally, it is recommended that randomized controlled clinical trials be conducted to evaluate the potential benefits of nutritional interventions in relation to physical growth, nutritional status and SC in the families of children and adolescents with SCA.
